# Adulteration of Cod-Liver Oil

**Published:** 1849-07

**Authors:** 


*Adulteration of Cod-liver Oil.*—To the Editor. Sir.—I must submit the following facts to you, in order that they may receive (if you think advisable) some notice in your Journal. From information which I have recently acquired, on a visit to several fishing places on our coast, I have abundant proof that ill-disposed persons are procuring oil from pollock, and other fish, for the purpose of offering it for sale in this city as pure cod-liver oil. Also that a concern in this city, who are dealers in whale, lard, and other oils, have stated that the use of cod-liver oil, as a medicinal agent, is all a humbug, and that they are "going in for making money while the excitement lasts;" and to favor appearances, occasionally procure a few cod-livers to blind the eyes of others. There are those, also, who, I fear, will, from their knowledge of mixing oils, resort to this deception, pretending to sell a pure article, because it is of handsome appearance—which is no proof of its genuineness, as the color will vary in the oil from different lots of livers, that are fresh and sweet, although in the spring and summer the oil will be somewhat darker in color than at other seasons. This I am convinced of, from personal experience for three years in the manufacture of the cod-liver oil. In the above statement, I do not implicate any druggist or apothecary in the adulteration of the oil. Respetfully yours,

*Boston, May,* 1849. Emery Souther.

*Boston Medical and Surgical Journal.*

